# Thin Central Corneal Thickness May Be a Risk Factor for Myopia Progression in Children

**DOI:** 10.1155/2023/3815863

**Published:** 2023-01-16

**Authors:** Peng Zhou, Dan-Dan Wang, Lei Fan, Lin Yang, Ming-Wei Zhao

**Affiliations:** ^1^Department of Ophthalmology, Parkway Gleneagles Medical and Surgical Center, Shanghai, China; ^2^Department of Ophthalmology, Shenton Health Hong Qiao Medical Center, Shanghai, China; ^3^Department of Ophthalmology, Visionly Plus Eye Hospital, Beijing, China; ^4^Shanghai Medical College, Fudan University, Shanghai, China; ^5^Department of Ophthalmology, Peking University People's Hospital, Beijing, China

## Abstract

**Purpose:**

This study investigated the correlation between corneal biomechanical parameters and the speed of myopia progression.

**Methods:**

This is a retrospective, multicenter study. Both Chinese and Caucasian children were involved. The follow-up time was at least 12 months. Ocular biometry data including the central corneal thickness (CCT), axial length (AL), corneal keratometry (K), anterior chamber depth (ACD), white-to-white (WTW) distance, and pupil size (PS) were measured. The age of onset, speed of progression of spherical equivalent (SE), and speed of AL elongation were calculated. Data were analyzed using the R programming language.

**Results:**

This study comprised 306 eyes of 153 myopic children. 122 children were Chinese, and 31 children were Caucasian. The myopia progression was faster in Chinese children than that in the Caucasian group in both SE progression speed and AL elongation speed. The CCT was negatively correlated with the SE speed of progression (correlation coefficient, *R* = −0.65, and *p*=7.25 × 10^−38^) and AL speed (*R* = −0.47 and *p*=1.62 × 10^−18^). CCT was positively correlated with the age of onset (*R* = 0.35 and *p*=4.53 × 10^−10^). No significant correlation (*R* > 0.3 and *p* < 0.01) was found between other ocular biometries (K, ACD, WTW, and PS) and the onset and speed of the progression of myopia. The same trends were found in both Chinese and Caucasian children and in both the right eye and left eye.

**Conclusion:**

CCT was negatively correlated with myopia (SE) progression speed and AL elongation speed. Thin CCT may be associated with faster myopia progression.

## 1. Introduction

Myopia is considered to be a significant public health problem worldwide [1, 2]. The prevalence of myopia in middle school-aged young adults is between 70% and 90% in the East and Southeast Asia [3, 4]. High myopia may cause cataracts, choroidal neovascularization, retinal detachment, glaucoma, and macular atrophy and may lead to significant irreversible visual impairment and blindness [5–7]. Therefore, preventing the progression of myopia is necessary.

Risk factor analysis could help identify the children who have a high risk of suffering from myopia and a high risk of fast progression of myopia. Ophthalmologists and optometry doctors may give those children early targeted intervention to delay myopia onset or slow myopia progression. Previous studies showed that education, limited time outdoors [8], sleeping late [9], smartphone use [10], race, parental myopia [11], birth order (first-born children) [12], and season of birth (born in summer) [13] are associated with myopia. However, many of the previously found risk factors are difficult to quantify. For example, limited time outdoors is strongly associated with myopia. However, children and their parents find it difficult to schedule exercise time for outdoor activities. In clinical practice, doctors prefer risk factors that can be accurately measured.

Ocular biometry involves anatomical measurements of the eye, including the central corneal thickness (CCT), axial length (AL), corneal keratometry (K) or corneal radius (CR), anterior chamber depth (ACD), white-to-white (WTW) distance, and pupil size (PS) [14]. Ocular biometry provides precise numbers. Previous studies found that AL/CR values are correlated with the spherical equivalent (SE) [15]. The correlation between CCT and myopia was controversial in the previous studies. Some doctors reported that CCT is correlated with the degree of myopia among adults [16]. However, no correlation between CCT and the degree of myopia was found among adults in Taiwan [17]. Furthermore, no studies investigated the association between CCT and the progression of myopia in children.

In this retrospective, multicenter study, we investigated the correlation between ocular biometry, especially CCT, and the progression of myopia in Chinese and Caucasian children in three eye centers. The purpose of this study was to determine whether thin CCT is a risk factor for myopia development among school-aged children.

## 2. Materials and Methods

### 2.1. Study Design and Subjects

This was an ophthalmic-center-based retrospective study. Three ophthalmic centers were involved in the study, namely, Parkway Gleneagles Medical and Surgical Center (PG), Shenton Health Hong Qiao Medical Center (SH), and Visionly Plus Eye Hospital (VP). PG and SH are international medical centers in Shanghai, which have both Chinese and Caucasian patients. VP only has Chinese patients.

The data collection period was from January 2020 to January 2022. The inclusion criteria for our subjects were as follows(i) age between 4 and 18 years, (ii) cycloplegic SE less than −0.5 diopters in the worst eye, (iii) lack of other serious eye diseases, (iv) ability of parents/guardians to provide informed consent, and (v) the follow-up time was at least 1 year (12 months).

### 2.2. Ethics Statement

The study adhered to the tenets of the Declaration of Helsinki. The approval for the study protocol was obtained from the Ethics Committee and Institutional Review Board of Parkway Gleneagles Medical and Surgical Center (Shanghai, China).

### 2.3. Examination

The technique used in this study was consistent across ophthalmologists in all three ophthalmic centers. Slit lamp examination with slit lamp lens (Digital Wide Field, Volk, USA) was performed by ophthalmologists to assess the anterior segment and posterior segment of the eyes. The myopia was measured twice for both eyes. After full cycloplegia by instillation of three drops of Mydrin-P (Tropicamide 0.5%, phenylephrine HCl 0.5%; Santen Pharmaceutical, Shiga, Japan) at 5 min interval and wait for 30 another minutes, autorefraction was performed with a desktop autorefractor (KR-8800; Topcon Corporation, Japan) at the baseline and repeated after 12 months. An optical biometry device (AL-Scan Optical Biometer, Nidek, Japan) was used to measure the CCT, AL, K, ACD, WTW, and PS after full cycloplegia. SE was calculated as sphere plus half cylinder. The data from both eyes of each subject were used in this study. No intervention (for example, orthokeratology or low-concentration atropine eye drops) was used.

### 2.4. Statistical Analysis

The speed of the progression of myopia = (SE at the end of follow-up − SE in the first follow-up)/(time of the last follow-up − time of the first follow-up) was calculated. The speed of AL = (AL at the end of follow-up − AL in the first follow-up)/(time of the last follow-up − time of the first follow-up) was calculated. The data were analyzed using the R programming language (version 4.1.3). Pearson correlation analysis was performed to analyze the relationship between the speed of the age of onset, SE, AL, and corneal biomechanical parameters. The prognostic nomogram for the risk of SE speed of progression was constructed using the R nomogram package. The significance level was set at *p* < 0.05.

## 3. Results

### 3.1. Study Sample Characteristics

This study comprised 306 eyes of 153 myopic children. 76 were males, and 77 were females. 122 children were Chinese, and 31 children were Caucasian. The patients' age of onset was in the range 4–17 years old. The mean age of onset, SE speed of progression, AL speed, K, ACD, CCT, WTW, and PS in different race groups are presented in Table 1. Sphere at the initial visit was −1.36 ± 1.07 Ds (range−0.50 Ds to −2.25 Ds), and the sphere at the last visit was −2.38 ± 1.35 Ds (range−0.50 Ds to −3.50 Ds). The cylinder at the initial visit was −1.12 ± 0.93 Dc (range−0.00 Dc to −2.00 Dc), and the cylinder at the last visit was −1.51 ± 1.39 Dc (range−0.00 Dc to −2.25 Dc). Five children (3.26%) had anisometropia (refractive error between eyes is over 1.00D). The correlations of ocular biometry between the right eye and the left eye are shown in Table 2.

Myopia progression was faster in Chinese children than in the Caucasian group in both SE progression speed (SE speed and *p*=0.002) and AL elongation speed (AL speed and *p*=0.020). Caucasian children's CCT (561.60 ± 3.14 *μ*m) was thicker than the Chinese group's CCT (543.74 ± 1.87 *μ*m and *p*=1.35 × 10^−5^). There was no statistically significant difference in the age of onset (*p*=0.069), corneal keratometry (*p*=0.212), ACD (*p*=0.669), WTW (*p*=0.428), and PS (*p*=0.689).

### 3.2. The CCT Was Correlated with the Onset and the Speed of Progression of Myopia

**Figure 1:**
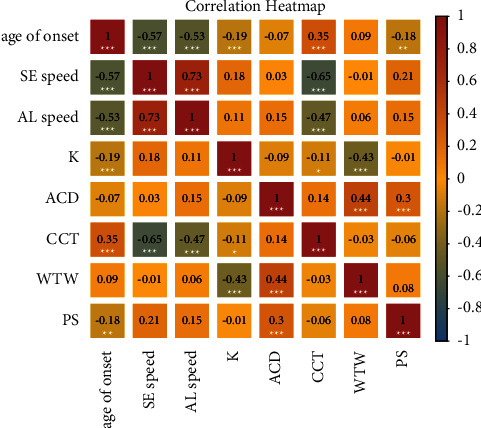
The correlation heatmap of the ocular biometries ((K) ACD, CCT, WTW, and PS) associated with the onset and speed of progression of myopia (age of onset, SE speed, and AL speed). The colors of the circles indicate the magnitude of the correlation between them Kcorneal keratometry, ACDanterior chamber depth, CCTcentral corneal thickness, WTWwhite-to-white, PSpupil size, ALaxial length, and SEspherical equivalent.

A heatmap of the correlations between the ocular biometries (K, ACD, CCT, WTW, and PS) associated with the onset and speed of the progression of myopia (age of onset, SE speed, and AL speed) was created (Figure 1). The CCT was negatively correlated with the SE speed of progression (correlation coefficient, *R* = −0.65 and *p*=7.25 × 10^−38^) and AL speed (*R* = −0.47 and *p*=1.62 × 10^−18^). The CCT was positively correlated with the age of onset (*R* = 0.35 and *p*=4.53 × 10^−10^). No significant correlation (*R* > 0.3 and *p* < 0.01) was found between other ocular biometries (K, ACD, WTW, and PS) and the onset and speed of the progression of myopia.

### 3.3. The Association between CCT and Myopia

**Figure 2:**
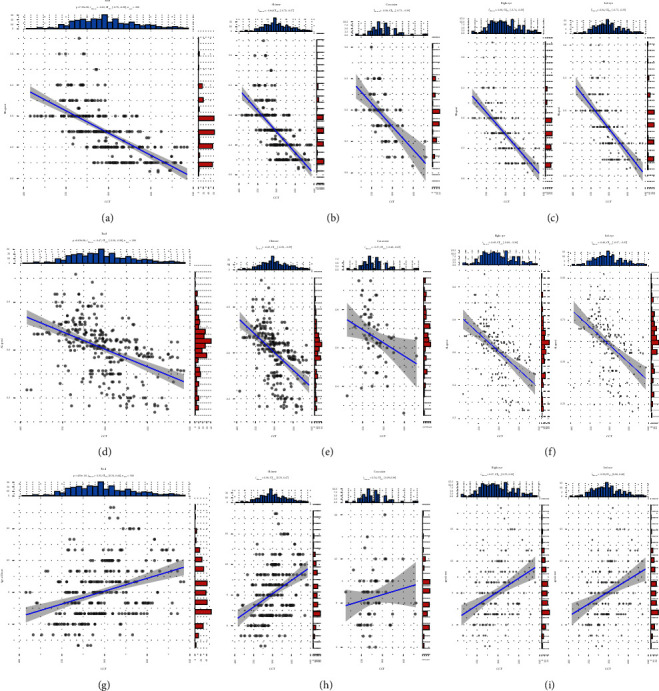
Scatter plots of the association between CCT and myopia. The CCT was negatively correlated with the SE speed and AL speed. The CCT was positively correlated with the age of onset CCTcentral corneal thickness, ALaxial length, and SEspherical equivalent.

The association between CCT and myopia is shown as scatter plots in Figure 2. The CCT was negatively correlated with SE speed of progression (Figure 2(a), *t* = −14.84, *p*=7.25 × 10^−38^, *R* = –0.65, 95% confidence interval [CI] = −0.71 to −0.58, and *n* = 306). Children with thinner CCT had faster SE speed of progression. A negative correlation was also found for the Chinese children (Figure 2(b), *t* = −13.15, *p*=3.65 × 10^−30^, *R* = −0.65, 95% CI = −0.71 to −0.57, and *n* = 244) and Caucasian children (*t* = −5.57, *p*=6.37 × 10^−7^, *R* = −0.58, 95% CI = −0.73 to −0.39, and *n* = 62) subgroups. Same trends were found in the right eye (Figure 2(c), *t* = −10.82, *p*=1.45 × 10^−20^, *R* = −0.66, 95% CI = −0.74 to −0.56, and *n* = 153) and the left eye (*t* = −10.12, *p*=1.08 × 10^−18^, *R* = −0.64, 95% CI = −0.72 to −0.53, and *n* = 153).

The CCT was negatively correlated with AL speed (Figure 2(d), *t* = −9.38, *p*=1.62 × 10^−18^, *R* = −0.47, 95% CI = −0.56 to −0.38, and *n* = 306). Children with thinner CCT had faster AL elongation speeds. A negative correlation was also found in the Chinese children (Figure 2(e), *t* = −8.76, *p*=3.58 × 10^−16^, *R* = −0.49, 95% CI = −0.58 to −0.39, and *n* = 244) and Caucasian children (*t* = −2.32, *p*=0.02, *R* = −0.28, 95% CI = −0.50 to −0.04, and *n* = 62) subgroups. Similar trends were found in the right eye (Figure 2(f), *t* = −6.94, *p*=1.10 × 10^−10^, *R* = −0.49, 95% CI = −0.60 to −0.36, and *n* = 153) and the left eye (*t* = −6.29, *p*=3.23 × 10^−9^, *R* = −0.46, 95% CI = −0.57 to −0.32, and *n* = 153).

The CCT was positively correlated with the age of onset (Figure 2(g), *t* = 6.44, *p*=4.53 × 10^−10^, *R* = 0.35, 95% CI = 0.24–0.44, and *n* = 306). Children with thinner CCT had early onset age of myopia. A positive correlation was found in the Chinese children (Figure 2(h), *t* = 6.18, *p*=2.68 × 10^−9^, *R* = 0.37, 95% CI = 0.26–0.47, and *n* = 244). However, no statistical significance was found in the Caucasian children (Figure 2(i), *t* = 1.16, *p* = 0.25, *R* = 0.15, 95% CI = −0.11 to −0.38, and *n* = 62) subgroup. Same tendencies were found in the right eye (*t* = 4.85, *p*=2.99 × 10^−6^, *R* = 0.37, 95% CI = 0.22–0.50, and *n* = 153) and the left eye (*t* = 4.23, *p*=3.98 × 10^−5^, *R* = 0.33, 95% CI = 0.18 to 0.46, and *n* = 153).

### 3.4. Nomogram for Predicting the Risk of SE Speed

**Figure 3:**
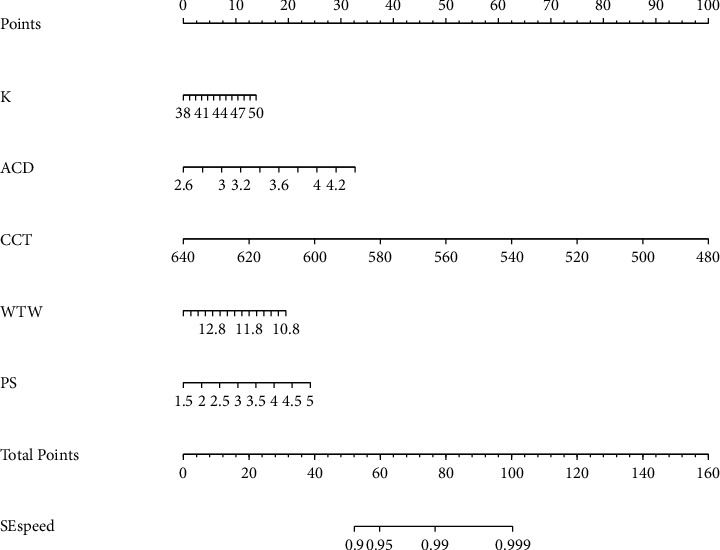
A nomogram model for predicting the risk of SE speed. We draw a straight line up to the point axis to determine the points assigned for each covariate. We sum the points and locate the total points on the bottom scale to determine the risk of SE speed of progression. The higher total points indicate a higher SE speed of progression Kcorneal keratometry, ACDanterior chamber depth, CCTcentral corneal thickness, WTWwhite-to-white, PSpupil size, and SEspherical equivalent.

The prognostic nomogram for the risk of SE speed of progression (Figure 3) was constructed based on the results of logistic regression analyses. The point of each factor can be determined by drawing a vertical line from the variable to the point axis. By summing up the total score and locating it on the total point scale, the estimated SE speed probability can be obtained.

## 4. Discussion

In this study, we found that the CCT was negatively correlated with the speed of myopia (SE) progression and AL elongation speed. Children with thinner CCT had faster speed of progression and AL elongation speed in both the Chinese and Caucasian populations. Moreover, the CCT was positively correlated with the age of onset. Children with thinner CCT had an early onset age of myopia.

One of the most commonly asked questions by parents in clinical practice is will their children's myopia increase quickly. It is difficult to answer this question because previously found risk factors, including education, time outdoors, and use of computers and smartphones, are difficult to quantify [8]. We can tell parents to let their children do more outdoor activities and use fewer screens. However, we cannot tell them the speed of the progression of myopia based on the previously found risk factors in the children's first visit. Ophthalmologists and optometrists need to follow the children for at least six months to one year to obtain the data and to predict the speed of myopia progression.

CCT can be measured during the children's first visit. A Nidek AL-Scan Optical Biometer was utilized to check the CCT. The CCT can be measured using ultrasonic and optical instruments. Previous studies suggested that the Nidek AL-Scan Optical Biometer device provides significant agreement with the ultrasound pachymetry and anterior segment optical coherence tomographer in the measurement of CCT [18–20]. An optical biometer does not contact the cornea; therefore, it is more suitable for checking kids' CCT than ultrasound pachymetry.

Most previous studies only investigated the power of myopia, not the speed of the progression of myopia, with the CCT in adults. Mimouni et al. reported that CCT is highly correlated with the degree of myopia among adults (*R* = 0.94) [16]. Touzeau et al. found that the central cornea was significantly thinner in highly myopic patients [21]. Srivannaboon found a statistically significant correlation between corneal thickness and the level of myopia [22]. Chang et al. suggested that corneas were thinner in more myopic eyes and the cornea tended to be thinner in eyes with longer ALs [23]. Von Bahr reported that myopia patients had thinner CCTs [24]. However, Ortiz et al. showed that there were no significant differences among low, moderate, and extremely myopic eyes related to the CCT [25]. Most of the previous studies only focused on myopia in adults whose myopia is stable. For children, the speed of the progression of myopia is a more important question.

In this study, we found that children with thinner CCT had faster speed of progression and AL elongation speed. The same tendency was verified in both Chinese and Caucasian children's subgroups. This correlation is pathophysiologically plausible. Corneal hysteresis is negatively correlated with axial elongation in children [26]. Corneal hysteresis is statistically significantly associated with CCT [27, 28]. This reasoning chain supports that the CCT is negatively correlated with myopia progression in children. Another explanation is that CCT is positively associated with scleral thickness [29]. The sclera is the final common pathway for visual signals to generate myopia progression [30]. Scleral remodeling is a crucial change in the development of myopia. Scleral remodeling makes the sclera thinner and makes axial elongation easier [31]. Thinner CCT suggests thinner sclera and indicates lower scleral stiffness [32]. Low scleral stiffness increases the risk of the progression of myopia [33]. Therefore, the thickness of CCT might be an indicator of the thickness of the sclera and an indicator of myopia progression speed.

Furthermore, the association between thin CCT and faster myopia progression provides an important basis for early interventions on myopia control. Doctors may suggest that children with thin CCT have shorter follow-up intervals, do more outdoor activities, and spend less time looking at screens.

The major limitation of this study was that the follow-up period was only one year. We will keep performing further studies with longer follow-up periods to confirm our findings. Furthermore, corneal ectasia will be assessed in our future prospective study.

In conclusion, the CCT was negatively correlated with speed of myopia (SE) progression and AL elongation speed. Thin CCT may be associated with faster myopia progression.

## Figures and Tables

**Table 1:** Study sample characteristics.

	Chinese (*n* = 244)	Caucasian (*n* = 62)	Between Chinese and Caucasian	Total (*n* = 306)
Age of onset	8.53 ± 0.17	9.19 ± 0.30	*p*=0.069	9.06 ± 0.15
SE speed	0.89 ± 0.03	0.71 ± 0.05	^*∗∗*^ **p**=0.002	0.75 ± 0.02
AL speed	0.65 ± 0.01	0.60 ± 0.02	^*∗*^ **p**=0.020	0.61 ± 0.01
K	43.22 ± 0.10	43.49 ± 0.17	*p*=0.212	43.27 ± 0.09
ACD	3.83 ± 0.02	3.81 ± 0.03	*p*=0.669	3.82 ± 0.01
CCT	543.74 ± 1.87	561.60 ± 3.14	^*∗∗∗*^ **p**=1.35 × 10^−5^	557.98 ± 1.67
WTW	11.97 ± 0.03	11.93 ± 0.05	*p*=0.428	11.96 ± 0.02
PS	3.52 ± 0.03	3.55 ± 0.06	*p*=0.689	3.52 ± 0.03

Mean ± standard error; SEspherical equivalent; ALaxial length; Kcorneal keratometry; ACDanterior chamber depth; CCTcentral corneal thickness; WTWwhite-to-white; PSpupil size; ^*∗∗*^*p* < 0.05; ^*∗∗*^*p* < 0.01; ^*∗∗∗*^*p* < 0.001.

**Table 2:** Correlation of ocular biometry between the right eye and the left eye.

	Pearson correlation coefficient (PCC) between the right eye and the left eye	*P* value
CCT	0.98	<0.001
K	0.98	<0.001
PS	0.86	<0.001
ACD	0.91	<0.001
WTW	0.86	<0.001
AL speed	0.96	<0.001
SE speed	0.94	<0.001

CCTcentral corneal thickness; Kcorneal keratometry; PSpupil size; ACDanterior chamber depth; WTWwhite-to-white; ALaxial length; SEspherical equivalent.

## Data Availability

The data used and analyzed during the current study are available from the corresponding author on reasonable request.
